# 替吉奥胶囊导致的急性间质性肺病1例报道

**DOI:** 10.3779/j.issn.1009-3419.2014.01.09

**Published:** 2014-01-20

**Authors:** 芳芳 李, 艳芳 鞠, 印 关, 宏 赵

**Affiliations:** 572000 三亚，中国人民解放军总医院海南分院 Department of Oncology, Hainan Branch of PLA General Hospital, Sanya 572000, China

替吉奥胶囊是一种氟尿嘧啶衍生物口服抗癌剂，临床应用时耐受性较好，用于胃癌、乳腺癌、非小细胞肺癌（non-small cell lung cancer, NSCLC）等多种恶性肿瘤的治疗。其主要剂量限制性毒性反应是骨髓抑制，肺损伤相对罕见，临床上仅有少数病例报道。日本学者曾报道数例服用替吉奥胶囊过程中并发间质性肺病（interstitial lung disease, ILD）病例，而国内尚未见报道。本文报道1例肺鳞癌患者病例，该患者在服用替吉奥胶囊后出现急性间质性肺损伤，并对相关文献进行复习。

## 1 临床资料

患者男性，42岁，身高：170 cm，体重：91 kg，体表面积：1.91 m^2^。继往吸烟30余年，每日吸烟约30支，发病后戒烟。2013年2月因“刺激性干咳半月余”到亳州市人民医院就诊，胸部CT示纵隔软组织占位（未提供影像资料，具体不详），气管镜活检病理为鳞状细胞癌，诊断考虑：右肺鳞癌，右肺门、纵隔淋巴结转移。2013年3月患者入我院给予1线第1-4周期DP方案化疗（多西他赛注射液，罗纳普朗克，75 mg/m^2^，140 mg，第1天）；注射用顺铂（冻干型）（75 mg/m^2^，140 mg，第1天），2周期后疗效评价为部分缓解（partial remission, PR），4周期后疗效评价为维持PR。考虑顺铂的肾脏蓄积毒性，4周期后决定给予替吉奥胶囊维持治疗（替吉奥胶囊，大鹏药品工业株式会，60 mg 1/早、60 mg 1/晚、连服14天休1天为1周期）。服用替吉奥胶囊2周后患者出现持续咳嗽，干咳为主，无痰，伴随间断发热（体温最高38.0 ℃），予以止咳、抗炎对症治疗症状缓解不明显，首次服用3周后患者按时给予第2周期替吉奥胶囊口服，咳嗽症状明显加重，故服用2天后停用替吉奥胶囊。因症状持续加重，于首次服用替吉奥胶囊4周后入院诊治。实验室检查示：白细胞计数6.19×10^9^L；中性粒细胞计数4.08×10^9^L；单核细胞1.09×10^9^/L；C-反应蛋白（C-reactive protein, CRP）：5.4 mg/dL；降钙素原0.07 ng/mL；血细菌培养、厌氧菌培养、真菌培养均阴性；甲型流感病毒检测阴性；肺炎支原体抗体检测阴性。心电图示窦性心律、心电图正常范围；胸部CT：双肺间质性渗出，肺泡间隔增厚，多发斑片状、片状磨玻璃密度影，考虑ILD（图 1A，图 1B），立即给予氧疗、激素治疗（注射用甲泼尼松龙琥珀酸钠，160 mg，每3天对半减量，减至20 mg后给予甲泼尼龙片20 mg维持2周，后逐步减量至停药）。给予注射用甲泼尼松龙琥珀酸钠后当天患者咳嗽症状明显缓解，未再出现发热。2周后复查胸部CT示双肺间质性渗出基本吸收（图 1C，图 1D）。该患者有高血压病史4年，长期服用硝苯地平缓释片，化疗期间服用利可君、鲨肝醇辅助骨髓保护，该类药物服用时间均较长，相关文献并无导致ILD报道。同时相关实验室检查除外了心脏疾病、感染诱发ILD，综合考虑患者为替吉奥胶囊诱发的ILD。

**1:**
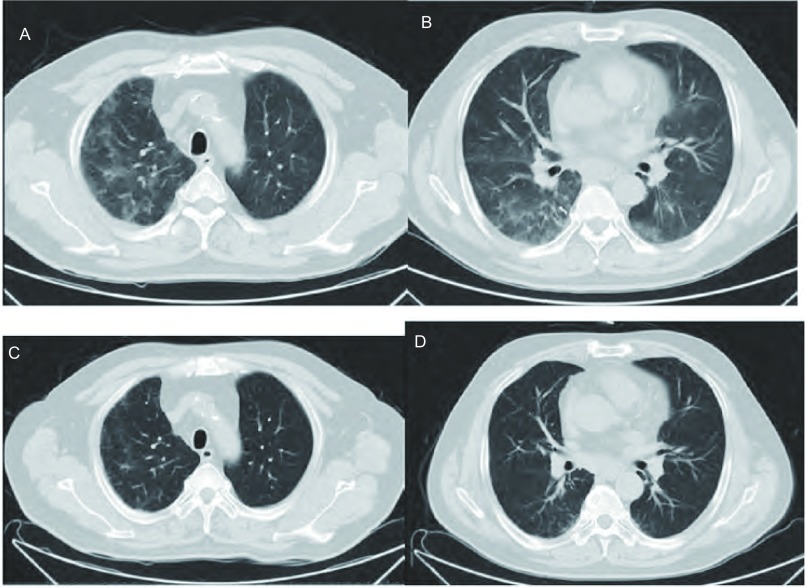
胸部CT检查结果。A、B：胸部CT示双肺间质性渗出，肺泡间隔增厚，多发斑片状、片状磨玻璃密度影；C、D：胸部CT示双肺间质性炎症较前明显好转。 Chest computed tomography findings on admission. Chest computed tomography (A, B) revealed diffuse interstitial lesions with thickening of the alveolar septa and multiple patchy, flake grinding glass opacity. Chest computed tomography (C, D) findings were substantially improved after initiating steroid pulse therapy.

该患者1月后复查，肿瘤进展，给予更换二线化疗，目前仍生存。

## 2 讨论

ILD是NSCLC常见的伴随疾病，且与肿瘤的治疗密切相关。在接受放疗、化疗以及靶向治疗的患者中，急性ILD的发生率甚至可高达10%^[1]^。ILD以弥漫性肺实质、肺泡炎和间质纤维化为基本病理改变，而药物诱发的ILD通常表现为弥漫性肺泡损伤，起病相对急促，严重影响预后甚至危及患者生命^[2]^。ILD的诊断主要依赖于临床症状和影像学表现^[3]^。临床表现主要为活动性呼吸困难、限制性通气障碍、弥散功能降低和低氧血症，影像学表现为双肺弥漫性阴影，阴影的性质可以是网格条索状、弥漫磨玻璃状、结节状。治疗上，药物性ILD并无针对性药物，以对症、支持治疗为主，首选撤药，多数患者撤药后症状会有所减轻；其次给予氧疗、改善通气以及糖皮质激素治疗，经过及时治疗多数患者可获得缓解。

准确诊断药物导致ILD的关键是明确药物与出现肺部症状和体征之间符合逻辑的时序关系^[4]^。虽然通常从开始服用药物到出现ILD的时间并不明确，但在应用该药物之前必须没有ILD相关的病史，相关症状也必须在服用药物之后出现。理想状态下，撤除诱因药物之后，与ILD相关的症状和体征也该停止继续进展。诊断的核心在于重新应用该药物会导致ILD的再次出现，但是这种诱发实验有较大风险，临床须慎重使用。临床诊断主要建立在排除性诊断的基础上，对于接受化疗药物者必须排除其他可能导致ILD的情况：感染、代谢性疾病、心脏疾病、癌性淋巴管炎以及肿瘤进展所致^[5]^。本例患者在服用替吉奥胶囊2周后开始出现咳嗽症状，并进行性加重，伴有间断发热，院外给予常规止咳、抗炎治疗，症状缓解不明显，再次给药后症状明显加重，首次服药4周后的胸部CT证实双肺间质性渗出，肺泡间隔增厚，多发斑片状、片状磨玻璃密度影，呈典型间质性炎症表现，相关检查排除了感染、心脏以及代谢问题，诊断考虑药物导致的ILD。治疗反应方面也符合ILD的特点，该患者糖皮质激素冲击治疗后症状即得到明显好转，激素治疗2周后胸部CT可见双肺磨玻璃样病变基本消失。

该患者此前应用多西他赛联合顺铂方案化疗4周期，既往文献曾有多西他赛导致ILD的相关报道。多西他赛所致ILD者多在接受多西他赛治疗1周-2周内出现急性呼吸困难和发热症状，易患人群为既往有ILD病史的患者^[6]^。该例患者既往接受过4个周期的多西他赛治疗，未出现肺损伤相关不良反应，4周期后疗效评价的胸部CT示肿瘤维持疗效PR，双肺野亦未见间质性渗出影。但在接受替吉奥胶囊治疗2周后出现症状，3周后再次尝试应用替吉奥胶囊，症状加重，提示替吉奥胶囊与ILD的关系更为密切。既往患者并无ILD相关病史，综合考虑用药情况及其与肺部症状出现的时序关系，推断替吉奥胶囊为ILD的病因。

替吉奥胶囊是由替加氟（Tegafur, FT）和两类调节剂吉美嘧啶（Gimeracil, CDHP）和奥替拉西（Oteracil Potassium, OXO）构成。替加氟是5-氟尿嘧啶（5-Fluorouracil, 5-Fu）的前体药物，能在活体内转化为5-Fu。CDHP能够抑制5-Fu的分解代谢，有助于长时间维持血中和肿瘤组织中5-Fu有效浓度。OXO能够阻断5-Fu的磷酸化，口服后在胃肠组织中具有很高的分布浓度，从而影响5-Fu在胃肠道的分布，进而降低5-Fu毒性的作用。通过上述药物的配伍，能够增加疗效，减轻不良反应^[7]^。替吉奥胶囊于2004年在日本被批准用于晚期非小细胞肺癌的治疗。在日本进行的2项替吉奥胶囊单药用于晚期非小细胞肺癌的二线治疗临床研究显示单药客观有效率在12.5%-14%，且无4度不良反应发生，提示单药替吉奥胶囊在晚期非小细胞肺癌治疗的有效性和安全性^[8]^。替吉奥胶囊的耐受性良好，剂量限制性毒性为骨髓抑制。在联合顺铂用于晚期NSCLC一线治疗时，3度-4度中性粒细胞减少发生率为18.3%，其他常见的不良反应包括恶心、呕吐、转氨酶升高、食欲减退、疲劳等，较多西他赛联合顺铂组要少，多数患者治疗耐受良好^[9]^。有关替吉奥胶囊肺毒性的发生相对较少，仅日本有少数病例报道^[10-16]^。Takiguchi等^[17]^设计了一项替吉奥胶囊用于铂类耐药的晚期非小细胞肺癌的临床研究，该研究入组既往使用过单药或双药含铂方案治疗失败的晚期非小细胞肺癌患者，接受吉西他滨联合替吉奥胶囊方案的治疗，共入组34例患者，中位化疗周期为3周期，有3例患者出现3度间质性肺损伤，1例出现2度间质性肺损伤，4例患者最终均恢复。本例患者是在替吉奥胶囊序贯多西他赛治疗的过程中出现ILD，提示替吉奥胶囊应用时特别是与吉西他滨、多西他赛等化疗药物联合应用时应密切注意急性肺损伤的发生。

药物可通过多种机制导致肺损伤：氧化/抗氧化系统、免疫系统、基质修复、蛋白酶/抗蛋白酶系统、干扰脂质代谢和中枢神经系统。药物可对肺产生直接或间接损伤作用。直接损伤可能通过毒性作用或者特殊的机制产生，间接损伤可能通过抑制中枢神经系统、免疫系统甚至造血系统对肺产生影响^[18]^。替吉奥胶囊导致ILD的原因也不明确，而既往通过药物淋巴细胞刺激试验研究发现ILD由替加氟产生的可能性较大^[12]^，且既往也有5-Fu、UFT（主要成份为替加氟）导致ILD的相关病例报道，具体的致病机制还需要进一步的研究证实。

虽然目前不能明确哪些接受化疗的患者是罹患ILD的高危人群，但回顾性病例研究也给了我们一些提示。继往文献报道和本例患者的汇总分析见表 1。这8例患者中，男性多于女性，男女比例为3:1，6例患者年龄 > 65岁，从服药到出现症状的时间多数在2个月内，但也有服药后2年出现ILD者，除1例患者疾病进展外，其余7例患者经过积极治疗，均得到了控制。Kudoh等^[19]^回顾性的研究了2, 551例接受化疗的肺癌患者罹患ILD的情况，在12周的随访时间内，未调整的发病率约为1.7/1, 000人-周，12周末的累积发病率为2.1%。主要的危险因素包括：高龄（> 55岁）、差的体力状况（≥2分）、吸烟病史、新近诊断非小细胞肺癌（< 6个月）、正常肺组织减少（CT扫面显示减少50%）、既往有慢性ILD病史、合并心脏疾病。

**1:** 文献报道替吉奥胶囊导致间质性肺炎病例 Reported cases of tegafur gimeracil oteracil potassium capsule induced interstitial lung disease

Ref.	Age (year)	Gender	Diease	Regimen	The latency from the 1^st^ use (d)	Outcome
Kurakawa^[10]^	70	Male	Gastric	S-1 single	36	Good/recovery
Shitara^[11]^	37	Female	Gastric	S-1 single	150	Poor/dead
Tada^[12]^	72	Male	Tongue	S-1 single	18	Good/recovery
Ueyama^[13]^	80	Female	Breast	S-1 single	5	Good/recovery
Yamamoto^[14]^	80	Male	Gastric	S-1 single	22	Good/recovery
Yamane^[15]^	66	Male	Pancreas	S-1 single	45	Good/recovery
Nohara^[16]^	75	Male	Colon	S-1 single+DDP	360	Good/recovery
Present case	40	Male	Lung	S-1 single	14	Good/recovery
S-1: tegafur gimeracil oteracil potassium capsule.						

综上所述，替吉奥胶囊越来越多的应用于多种肿瘤，ILD也是其可能伴随的不良反应，特别是对于年龄较大、吸烟等高危人群，临床需予以密切关注。
